# Traveling Letter, No. IX

**Published:** 1844-12

**Authors:** 


### THE WESTERN JOURNAL.

NEW SERIES.—VOL. II.—NO. VI.

LOUISVILLE, DECEMBER 1, 1844.

### TRAVELING LETTERS FROM THE SENIOR EDITOR.

### NO. IX.

GALENA, Ill., Oct. 7th, 1844.

*To my Colleagues:*

GENTLEMEN:—Having in a journey of nearly 200 miles traversed the territory of Wisconsin, and reached the emporium of the 'Mineral Region,' I sit down to give you some account of it.

In passing up the Illinois river, I indicated to you the northern limit on that stream, of the great coal basin of the states of Indiana, Missouri, and Illinois, about the latitude of 41° 30′. Beyond that parallel there appears a lime stone, emerging from beneath the coal field, which in its mineralogical and fossiliferous characters, seems to be nearly identical with that which crops out to the south-east above and below Louisville. It is in this rock that the lead ore is found; but all parts of it around the coal basin are not metalliferous. Indeed, the extensive out-cropping from the middle part of the valley of Rock River, round through Illinois, Indiana, Ohio, and Kentucky, down to the mouth of the Wabash, seems to be destitute of metals; while that from the river last mentioned, round through southern Illinois. eastern Missouri, south-eastern Iowa, south-western Wisconsin, and north-western Illinois, affords lead and some other metals. But all parts of this semi-circular belt are not equally prolific. The lead mines back of Shawnoetown, southern Illinois, are not worked; in Missouri the most productive are in the rear of St. Genevieve, south-west of St. Louis, where iron is still more abundant than lead; in Iowa the most valuable are in the neighborhood of Dubuque, not far below Prairie du Chien; in Illinois, Jo. Daviess, the north-west county, and in Wisconsin, Grant and Iowa, with a portion of Dane, are those in which the greatest quantity of that metal has been found. It is the region then, composed of adjoining and continuous portions of Illinois, Wisconsin, and Iowa, in which I am, and of which I propose to speak, under the popular title of the

*'Mineral Region.'*

This tract, on the east side of the Mississippi larger than that on west, and the only part which I have visited, is drained by streams which flow into the Wisconsin on the north, and into the tributaries of the Platte and Fever river, which run to the south-west, and discharge their waters into the Mississippi. Its general level above Lake Michigan cannot be much less than 500 feet, or about 1,100 feet above the level of the sea. Its surface is undulating, and in some portions rather hilly. Scattered over it, are a number of insulated conoidal hills, known under the names of the Blue, Platte, and Sissinawa Mounds, one of which was ascertained by Professor Locke to rise 1748 feet above the level of the Ocean. These mounds would seem to indicate the ancient level of this region; and offer a satisfactory explanation of the absence of erratic blocks, bowlders and gravel on this surface, while they exist in countless numbers, east of this tract to the sources of the Allegheny river; and also to the south, on the lower levels of Rock and Illinois river. When these rocky fragments were rolled, or, imbedded in ice, were floated down from the north, the Mineral region was too elevated to receive them. This region abounds in copious perennial springs, and is everywhere covered with a fertile soil, thus presenting the unusual association of a productive agricultural surface with rich metalliferous rocks below. These rocks, like those of Kentucky, abound in rents, chasms, and caves, though of the last none of much extent have yet been discovered. It is in these interstices, that the ores are deposited. The search after them is commenced by digging perpendicularly from the surface; and the counties of Iowa and Grant in Wisconsin, and of Jo. Daviess in Illinois, present an incredible number of these perforations, a very large majority of which have been failures; and while they show that the search after ore, very often ends in disappointment, they are to a certain extent a nuisance, for cattle frequently fall into them and perish. The grass of the prairies and open woodlands, has grown over the banks of earth thrown out from many of these abandoned excavations, so that even men are liable to walk into the older ones. When the miner strikes upon a vein of ore, or, in his techincal language, a 'lead,' he makes new excavations at short distances, in the course of the fissure, for his convenience in raising the ore. These fissures are generally perpendicular, but sometimes the crevices will take a horizontal direction, and the ore is then said to be in beds. The vein is sometimes full, and the ore adherent to the rocks, or even blended with them; and may vary in thickness from a mere plate or film to a foot or more; but many crevices are partly filled with sand or loam, containing abundance of oxide of iron, in the midst of which are detached fragments and blocks of ore. In some of the larger chasms or small caves, masses of ore so large as to require blasting with gun powder, have been discovered. I need scarcely tell you that the metal of which I speak is LEAD, as this region is known abroad by that metal chiefly. It is galena or the sulphuret only, that is smelted. Much of this is quite amorphous, but cubes of various sizes, solitary or so aggregated, that but a part of the sides and angles of each can be seen, are common. Octahedrons are also met with. On the whole, however, in searching for specimens for the Cabinet of the Institute, I found them less numerous than I had expected. Rhomboidal calcareous spar, sulphate of barytes, and fluate of lime, are not wanting, but much less common than in the mines of St. Genevieve and Shawnoetown. Carbonate of lead is occasionally met with, but has not yet been smelted.

As you would expect, sulphuret of zinc, or blende, is found in connexion with the lead ore, but as yet has been thrown aside. Preparation is making, however, for the reduction of this ore, and the manufacture of zinc; which will undoubtedly be useful in a national point of view, but whether profitable to those who may undertake it, remains to be seen.

Copper ore has likewise been discovered. It is in nearly the same localities with the lead, and found in similar crevices. Two furnaces have been erected for smelting it, one of which near Mineral Point, Iowa county, I visited and obtained specimens for our Cabinet.

Iron ore is common in this region, but does not, as far as I have heard, occur in large quantities and has not yet been worked.

Lastly, Bismuth has been discovered and other metals will no doubt be brought to light.

Not going largely into the natural history of these ores, I shall go still less into their metallurgy. The smelting of the lead ore, is abundantly simple. The miner sells it to the smelter, who washes it in running water, and then crushes it between cast iron rollers, when it is thrown into a cast iron furnace, and by means of a fire of wood and charcoal, urged on by a bellows worked by water-power, the volatile ingredients of the ore are driven off, the earthy concrete into slag, and the melted lead flows out to be cast into 'pigs.' The slag contains a portion of lead, and is smelted over. No flux, or only a little lime is used. The chimnies of the furnaces become lined with a whitish crust, which is composed, it may be presumed, of carbonate of lead, with a mixture perhaps of arsenic.

The copper ore after ablution is broken by hand with a hammer, and subjected to the heat of a bellows furnace, with fluxes. The crude metal flows into a basin-shaped cavity in the hearth, and is taken out in cakes which are fragments of solid spheres. It requires to be refined before before being used by the artist.

The lead ores no doubt contain silver, but the proportion, I believe, has not been thought sufficient to warrant its extraction.

The mines of this region were at first worked by American backwoodsmen, who understood how to kill deer or plant corn, much better than to mine. At present, almost all the operatives are from Cornwall, England, and thorougly acquainted with their business. They have successfully re-opened many veins which had been abandoned, profitably smelted banks of slag which had been neglected, and so improved the construction of the furnaces, as to carry off the fumes which formerly enveloped the smelters. Their small one story cottages, many of which have their roofs covered with sods, which still continue to send up grass and flowers, are scattered over the prairies, and look not unlike the cabins of the Shawanoes, in the Indian Territory. It is not known, what number of persons inhabit these humble tenements, but the amount of population must be considerable; as Mr. Melvill, the Government Agent, informs me, that 40,000,000 pounds of lead were manufactured in this region last year, which is nearly one-half of the produce of all the mines of Great Britain. A small part of the mineral lands have been sold by the Government to individuals, the rest are let on leases, or worked by squatters. The Government claims six per cent. of all the lead manufactured, except by those who hold their lands in fee simple, but the amount collected is scarcely sufficient, I believe, to pay the salary of the Agent; who informed me that of the 12 or 1400 lessees, between 2 or 300 only, paid the Government any thing; and they returned any quantity they chose, as the annual amount of their raising. Within the mineral region no land can be purchased but on the oath of the applicant that he has examined it, and does not know that there is ore beneath. This temptation to perjury is irresistable, and no government should continue it. An individual is sometimes led blind-folded over a tract, on which others acting in collusion, had discovered ore; he then applies at the land office, and swearing that he had been over the tract and *saw* no 'mineral,' becomes its nominal owner for the benefit of the company! Again, freeholders within the district, may sell the ore of lessees or squatters, as obtained from their own lands. I concur with every individual with whom I have coversed, in the opinion, that the Government should abandon the present policy, and sell the 'mineral' lands to the highest bidder, making five dollars an acre the *minimum* price. It has not, and never will derive a revenue from them, while it retains the fee simple. They are becoming exhausted, at least in their superficial veins, by lessees and intruders who pay nothing, and after a time they will sell for even less, than lands of the same fertility, which never had any metalic treasures.

I am surprised to find, that in the office of the Government Agent, there is no collection of specimens—no geological sections—no descriptions;—but am happy to make known, to such of our readers as may visit Galena with a mineralogical taste, that they may find a 'curiosity shop,' by calling on a Justice of the Peace!—John G. Potts, Esq., formerly of Philadelphia, where, as an amusement, he acquired the rudiments of mineralogy, has made an extensive collection of the ores and fossils of this region, and very obligeingly exhibits them to all travelers who call upon him. It was in his cabinet, scientifically arranged and labelled, that I saw the only specimens of sulphate of barytes, fluate of lime, carbonate of lead, and bismuth, which I have met with in this quarter. He has likewise all the varieties of iron, zinc, and copper ores found hereabouts.

Among his fossils, which are substantially the same with those of the limestone formations of eastern Indiana, Ohio, and Kentucky, I saw the cast of a univalve shell in sulphuret of lead. But the most unexpected exhibition, was that of the head of the thigh bone with its socket, of the Mastodon, taken from a small cavern with nodules of lead ore, more than forty feet beneath the surface. In reaching it, the miners had to blast some of the superincumbent rocks. The animal had no doubt fallen into this chasm through an opening since filled up. Mr. Potts will be happy to exchange the minerals and fossils of this region for those of other places, but having sent off three boxes, without receiving anything in return, has very reasonably determined, hereafter to exact payment in advance. With this advertisement, I shall close my notice of the mines, lest you should suppose that I fancy myself a *naturalist;* and to attone to our readers, for so much of what they may regard —and justly too—as extra professional, I will give them a paragraph on the diseases of those who work in lead.

Most of the miners are addicted to Saturday afternoon and Sunday drinking, but the extent to which they carry it, is much less than formerly. Their diet is simple, consisting chiefly of bread and meat, though they might have milk, and potatoes or other vegetables in abundance; but throughout the mining district agriculture is sadly neglected. Those engaged in drawing up the ore, and in washing it, are much exposed to the weather, but they who excavate the mines, work both in summer and winter, in a temperature of about 50°, with a humid atmosphere. I had expected to find them peculiarly subjected to rheumatism, but while some of the medical gentlemen of the district thought them so, others thought not. They are not, I believe, more subject to pulmonary affections than the other inhabitants of this region. The handling of the ore does not seem to be the source of any malady. But the *smelters* are more or less affected with *colic.* Several years ago that disease was more prevalent than at present. The furnaces then, as I have already stated, were of such a rude construction, that the smoke and vapors were not adequately carried off, and their inhalation by the operatives produced many cases of colic, some of which proved fatal. The improved construction of the furnaces of the present day, has greatly abated the frequency of that affection. The operatives, moreover, practise greater ablution than formerly and many of them rinse their mouths before eating, by which the surfaces of the body have the deposits of lead carried off. As a preventative, oils and fat meats are said by the medical gentlemen here, not to be without efficacy. The 'hand' who can eat the greatest quantity of fat meat, is said to be most exempt. Some of the proprietors furnish olive oil, of which a tablespoonful is taken every morning; others substitute castor oil, believing that all the benefit is derived from an aperient effect. As the symptoms and treatment of the colic, here, are the same as elsewhere, I need not narrate them, but may mention that Dr. Crawford subjected the alvine excretions of one patient to the action of heat, and that they afforded globules of lead. Dr. Newhall informs me, that he has observed insanity to be more frequent in Galena and its neighborhood, than he has ever known it elsewhere. He can recollect six cases within the last year, which is certainly a very great number for the amount of population.—This frequency, if its reality be admitted, is not to be ascribed to lead, but to the excitement connected with the search after its ores; which is, indeed, very great, and extends to a large proportion of the people. Now and then, a vein is hit upon, which proves almost a little fortune; but hundreds of abortive examinations bring depressing, and sometimes ruinous disappointment. The mines are of great value to the people of the United States, but as a source of revenue to the Government we have already seen they are worthless; and the aggregate property of all who have engaged in working them, is, I have no doubt, less than it would have been, if they had expended the same labor and capital in cultivating the fertile soil which overspreads the veins of metal.

FEVER RIVER, October 8, 1844.

I beg of you not to be alarmed by the date of this continuation, for protentous as it seems, the people on its banks are as free from fever as their neighbors. Fever River originates in Wisconsin, and passing its southern boundary, enters the Mississippi 10 or 12 miles below Galena. It is navigable up to that town, whenever the Missouri is navigable, but no further on account of rapids. Its alluvions are narrow, and the lime stone hills which rise on each side, are abrupt and rugged. Between and upon these hills, is the flourishing town of Galena, whose name indicates its character, much more correctly, than the character of the river is expressed, by its singular appellation. As a medical traveler, I feel that I have a prescriptive right to investigate the origin of this title, certainly the least enviable, ever borne by a rocky, little river, whose limpid waters are the tribute of a thousand perenial springs. But to leave the florid for the etymological, I proceed to say, that the oldest authority to which I have access, (*Chandler*, 1829,) informs us, that the *Sacs and Foxes* gave to this stream the epithet *Macawbie*, which in their language signifies small-pox, or a fever with blisters; and that the name under consideration, is but an imperfect translation from their vocabulary. He proposes to adhere to the untranslated word, but as the world delights to perpetuate a bad name, his advice has not been followed. This etymology, however, is denied by *Peck*, 1834, who on the authority of another tradition, derives the name from M. Le Fevre, a French trader, who resided near its mouth. Finally, another tradition still, forms it out of *feve*, (bean river,) perhaps, from the excellent flavor of the beans, which some industrious squaw may have cultivated on its banks. Contenting myself with having embodied these data, I shall not presume to decide a question of so much delicacy, but submit it to the professional etymologist, with the concluding remark, that *Lapham*, 1844, concurs with *Peck*, and that all the assigned derivations, very justly exclude the idea, which the present appellation suggests to the mind.

I am now embarked for home, and the deep autumnal dies of the hills, in which this quiet little water is embosomed, remind me, that to be at my post in the Institute, in due time, I must reserve for a future visit, the young towns which adorn the banks of the Mississippi between this and St. Louis.

Your very ob't. serv't.

